# Genome-Wide Association Mapping of Anther Extrusion in Hexaploid Spring Wheat

**DOI:** 10.1371/journal.pone.0155494

**Published:** 2016-05-18

**Authors:** Quddoos H. Muqaddasi, Ulrike Lohwasser, Manuela Nagel, Andreas Börner, Klaus Pillen, Marion S. Röder

**Affiliations:** 1 Department of Breeding Research, Leibniz Institute of Plant Genetics and Crop Plant Research (IPK-Gatersleben), 06466, Stadt Seeland, Germany; 2 Institute of Agricultural and Nutritional Sciences, Martin Luther University Halle-Wittenberg, 06120, Halle, Germany; Institute of Genetics and Developmental Biology, CHINA

## Abstract

In a number of crop species hybrids are able to outperform line varieties. The anthers of the autogamous bread wheat plant are normally extruded post anthesis, a trait which is unfavourable for the production of F1 hybrid grain. Higher anther extrusion (AE) promotes cross fertilization for more efficient hybrid seed production. Therefore, this study aimed at the genetic dissection of AE by genome wide association studies (GWAS) and determination of the main effect QTL. We applied GWAS approach to identify DArT markers potentially linked to AE to unfold its genetic basis in a panel of spring wheat accessions. Phenotypic data were collected for three years and best linear unbiased estimate (BLUE) values were calculated across all years. The extent of the AE correlation between growing years and BLUE values ranged from r = +0.56 (2013 *vs* 2015) to 0.91 (2014 *vs* BLUE values). The broad sense heritability was 0.84 across all years. Six accessions displayed stable AE >80% across all the years. Genotyping data included 2,575 DArT markers (with minimum of 0.05 minor allele frequency applied). AE was influenced both by genotype and by the growing environment. In all, 131 significant marker trait associations (MTAs) (|log_10_ (*P*)| >FDR) were established for AE. AE behaved as a quantitative trait, with five consistently significant markers (significant across at least two years with a significant BLUE value) contributing a minor to modest proportion (4.29% to 8.61%) of the phenotypic variance and affecting the trait either positively or negatively. For this reason, there is potential for breeding for improved AE by gene pyramiding. The consistently significant markers linked to AE could be helpful for marker assisted selection to transfer AE to high yielding varieties allowing to promote the exploitation of hybrid-heterosis in the key crop wheat.

## Introduction

Thanks to the phenomenon of heterosis, F1 hybrid cultivars in a number of crop species are able to out-perform line varieties, both with respect to economic yield and to yield stability [1–5]. Although attempts to exploit hybridity for bread wheat improvement date back many years [6,7], still the market share of hybrid cultivars remains very small [2]. The slow development of hybrid wheat breeding, unlike its rapid adoption in other crops, notably maize, reflects largely practical difficulties associated with the production of hybrid grain, which arise from the strongly cleistogamous nature of wheat's breeding system [4]. To obtain a reasonable yield of hybrid wheat grain, the female parent needs to be not just male sterile, but its flowers must open sufficiently while the stigma is still receptive in order to allow access for incoming pollen; meanwhile, the male parent, rather than shedding its pollen within the closed floret, must extrude its anthers prior to anthesis. Consequently, the greater the extent of anther extrusion (AE), the greater will be the rate of cross fertilization for better hybrid seed production. Although, male sterility can be effectively induced either via treatment with a gametocide or by genetic means [4], some remodelling of floral architecture is required to ensure a sufficient volume of viable pollen [7,8].

AE like the majority of agronomically important traits, is typically under polygenic control [9,10]. The genetic basis of such traits has been most frequently uncovered through a quantitative trait locus (QTL) mapping approach, based on segregation patterns exhibited by a bi-parental mapping population. A more recently developed method, termed “association mapping” (AM), attempts to discover associations between phenotype and genotype in a panel of non-related germplasm [11–13]. AM offers three specific advantages over the conventional QTL mapping technique, namely an increased mapping resolution, a smaller investment in research time and cost necessary for the development of bi-parental mapping populations, and an opportunity to survey a wider range of alleles at any given locus [14]. Here, AM was carried out to identify the genetic basis of AE in a panel of spring wheat cultivars. The DArT system [15] was chosen as the marker platform, as it readily generates medium to high numbers of informative, genome-wide markers in a multiplexed format. Recent studies by employing DArT marker genotypes for GWAS analyses support the utility of this platform for sufficient genome-wide coverage and efficient MTA analysis in wheat [16–19].

## Methods

### Plant materials and phenotyping

The panel of 111 spring wheat accessions, maintained by the IPK-Gatersleben Genebank (http://www.ipk-gatersleben.de/en/genebank/), comprised 57 accessions bred in Europe, 31 in Asia, ten in each of North and South America and one in Australia; two accessions were of unknown origin (S1 Table). The investigated wheat accessions were mainly released in the 1970’s. The set was field-grown in Gatersleben, Germany (51°49′N, 11°17′E, 112 m asl) in three consecutive years (2013, 2014 and 2015), arranging the accessions as a randomized complete block design (four replicates in 2013 and 2014, but only one in 2015). The individual plot size was 1 x 1.5 m, and each plot was split into four rows spaced 0.2 m apart. Standard agronomic wheat management practices were applied. No specific permissions were required for the field experiments conducted in this study and no endangered or protected species were involved. Anther retention (number of non-extruded anthers) was scored in 2013 and 2014 by observing the anthers retained inside the four pairs of primary and secondary florets sampled from the central portion of four spikes per plot. In 2015, ten spikes per plot were harvested 5–10 days post anthesis and held at -20°C until analysis. Anther retention was then scored after the spikes had been returned to room temperature. AE was calculated by subtracting the number of retained (non-extruded) anthers from 24 (since the total number of anthers housed by eight florets is 24).

### Statistical analysis

The mean AE in each growing year was used to calculate the best linear unbiased estimate (BLUE) values, assuming fixed genotypic effects. The calculation was performed by routines implemented in GenStat v16 software (VSN International, Hemel Hempstead, Hertfordshire, UK) applying the “Mixed models REML” module and the “Linear mixed models” function. A Pearson correlation coefficient among the three growing years was also calculated. The repeatability among the biological replicates was calculated from the expression *σ*^*2*^*g* / (*σ*^*2*^*g* + *σ*^*2*^*e* / n_r_). The broad sense heritability of AE was calculated from the expression *σ*^*2*^*g* / (*σ*^*2*^*g* + *σ*^*2*^*g*._*E*_*/ n*_*E +*_ *σ*^*2*^*e* / n_E_.n_r_), where *σ*^*2*^*g* represented the genetic variance, *σ*^*2*^*e* the environmental variance, n_E_ is the number of environments and n_r_ the number of replicates.

### Genotyping and population structure

DArT profiling was performed by Triticarte Pty. Ltd. (www.triticarte.com.au); the initial number of scored dominant loci was 2,836. An allele frequency minimum threshold of 5% was applied prior to the determination of marker-AE associations which reduced the size of the data matrix to 2,575 x 111. The quality of these markers was high, with an average call rate, reproducibility and polymorphism information content value as 96.37%, 99.53% and 0.36, respectively.

In order to determine the appropriate population structure and for the assessment of the number of sub-groups in the whole panel, a Bayesian model based approach implemented in the software package Structure v2.3.4 was performed [20]. This method attempts to assign individuals to populations (K) on the basis of their genotypes without prior knowledge of their population kinships and assumes that the loci are at linkage equilibrium and at Hardy-Weinberg equilibrium within populations. The program was run with polymorphic DArT markers with a minor allele frequency (MAF) >5%, assuming K = 1–20 with 100,000 burn-in iterations followed by 100,000 MCMC (Markov Chain Monte Carlo) iterations for accurate parameter estimates. We performed 10 independent runs for each K and calculated their average to further validate the results. An admixture model with correlated allele frequencies was used. Structure Harvester [21] was used to summarize the repeated runs of Structure v2.3.4, which determined the optimal K by using the Evanno method [22]. To further summarise and compare the results, we used CLUMPAK [23], which generated figures with individual assignment probability to each K cluster using consistent colouring of each cluster.

Genetic relationships amongst the spring panel accessions were also investigated graphically via principal coordinates analysis (PCoA) based on DArT genotype with the software package NTSYSpc (http://www.exetersoftware.com/cat/ntsyspc/ntsyspc.html). The first two principal coordinates were graphed in two-dimensional space to show the clustering of different accessions.

The Loiselle kinship matrix derived from the full set of DArT markers (MAF >5%) was generated using the SPAGeDi-1.3d program [24].

### Association mapping and linkage disequilibrium assessment

Marker trait associations (MTAs) involving AE and the DArT markers were identified using the “QTL analysis” module and the “Single trait association analysis” function implemented in GenStat v16, applying a Loiselle kinship matrix (K) for the purpose of correction for population stratification. Thus each AE value was modelled by the expression μ + marker effect + genotype effect + residual error, where the genotype effect was assumed to be normally distributed with *~ N* (*0*,*2Kσ*^*2*^_*genotype*_), also the residual error was assumed to be normally distributed with *~ N* (*0*, *σ*^*2*^_*e*_). The threshold of |log_10_ (*P*)| ≥2.0 was set in GenStat v16 for the detection of significant markers. However, this set of DArT markers was subjected to another test of robustness using the false discovery rate (FDR) at 0.05 [25]. The detection of the markers >FDR was estimated with the help of R-package q-value [26]. Thus, the markers which had |log_10_ (*P*)| >FDR in different years and BLUE values individually were considered as significant markers. The markers which appeared significant (|log_10_ (*P*)| >FDR) across at least two years and BLUE values, were termed as consistently significant markers. |log_10_ (*P*)| value distributions were displayed in the form of Q-Q plots for each growing year and across years (using BLUE values). For the set of markers unlinked to an AE QTL, the |log_10_ (*P*)| values would be expected to be uniformly distributed, and large deviations are indicative of spurious associations [27,28].

TASSEL v3.0 was used to calculate the phenotypic variance imparted by each marker (R^2^). The additive contribution (effect) of each linked marker was calculated with GenStat v16: negative effects reflect a decrease in AE (unfavourable alleles), and positive ones an increase (favourable alleles). A partial linkage map was constructed with the significant markers (|log_10_ (*P*)| >FDR) having known map location and non-significant markers ensuring even genome coverage (map positions taken from Triticarte Pty. Ltd., and Detering et al. [29]); this was drawn using MapChart 2.2 software [30].

Using the set of mapped DArT markers genome-wide linkage disequilibrium (LD) was studied in the whole panel of spring wheat accessions. Intra and inter-chromosomal quantification and graphical representation of LD heat maps and intra-chromosomal LD decay was accomplished with GenStat v16.

## Results

### Phenotypic analysis and heritability

**Fig 1:**
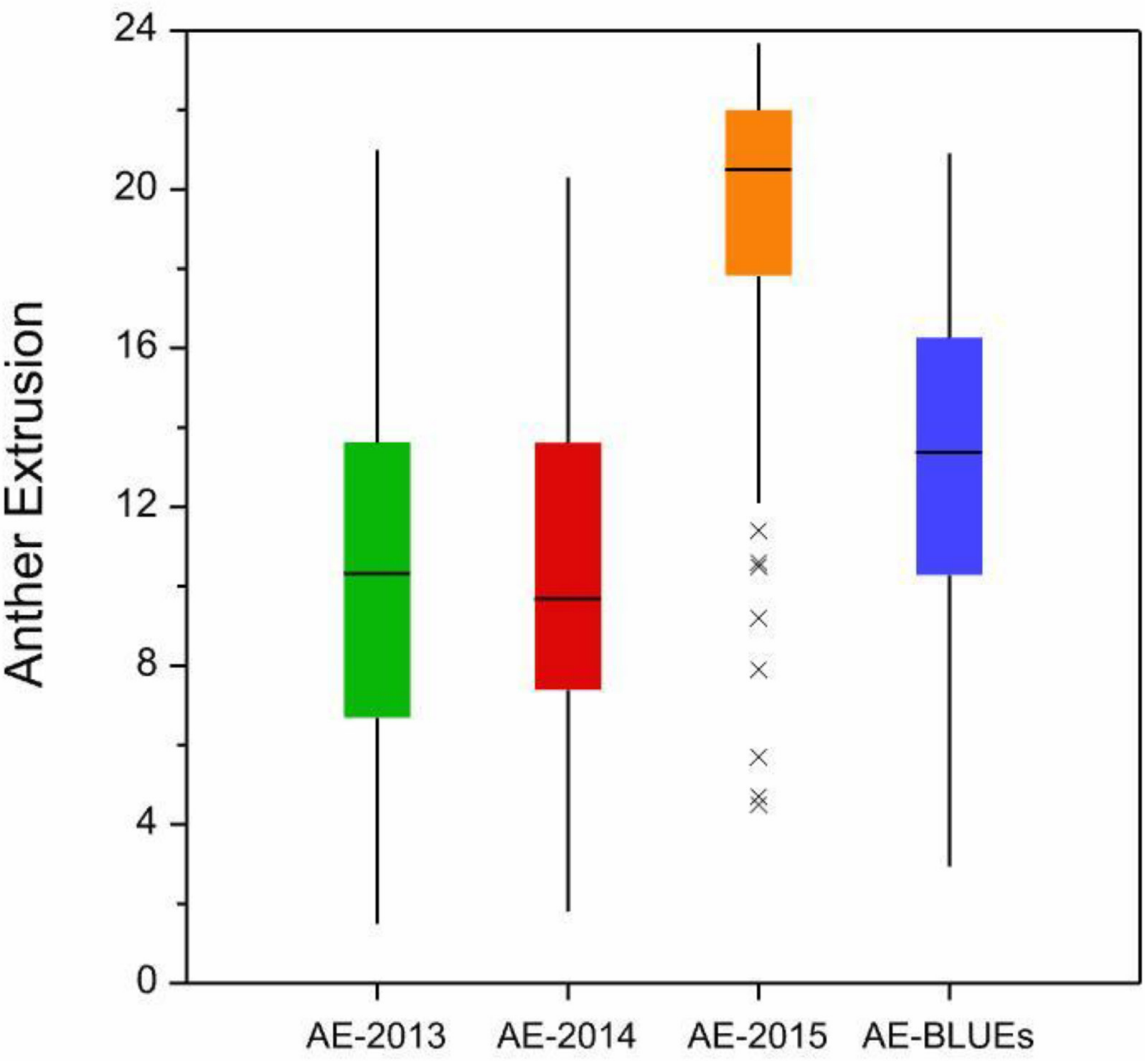
Boxplots displaying distribution of AE in the three years and BLUE values. Asterisks indicate outlier accessions.

**Fig 2:**
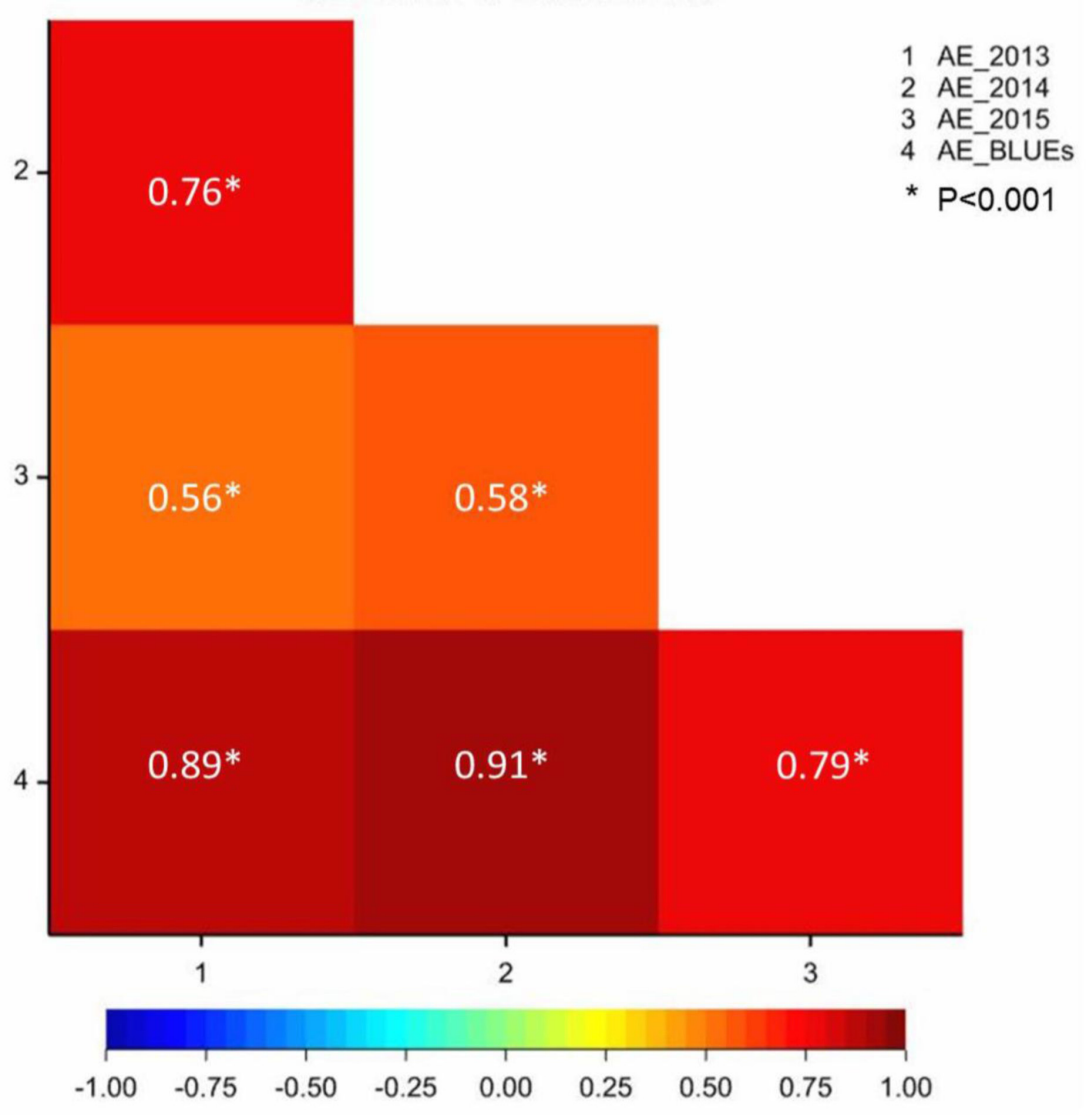
Pearson correlation coefficients among growing years and BLUE values.

**Fig 3:**
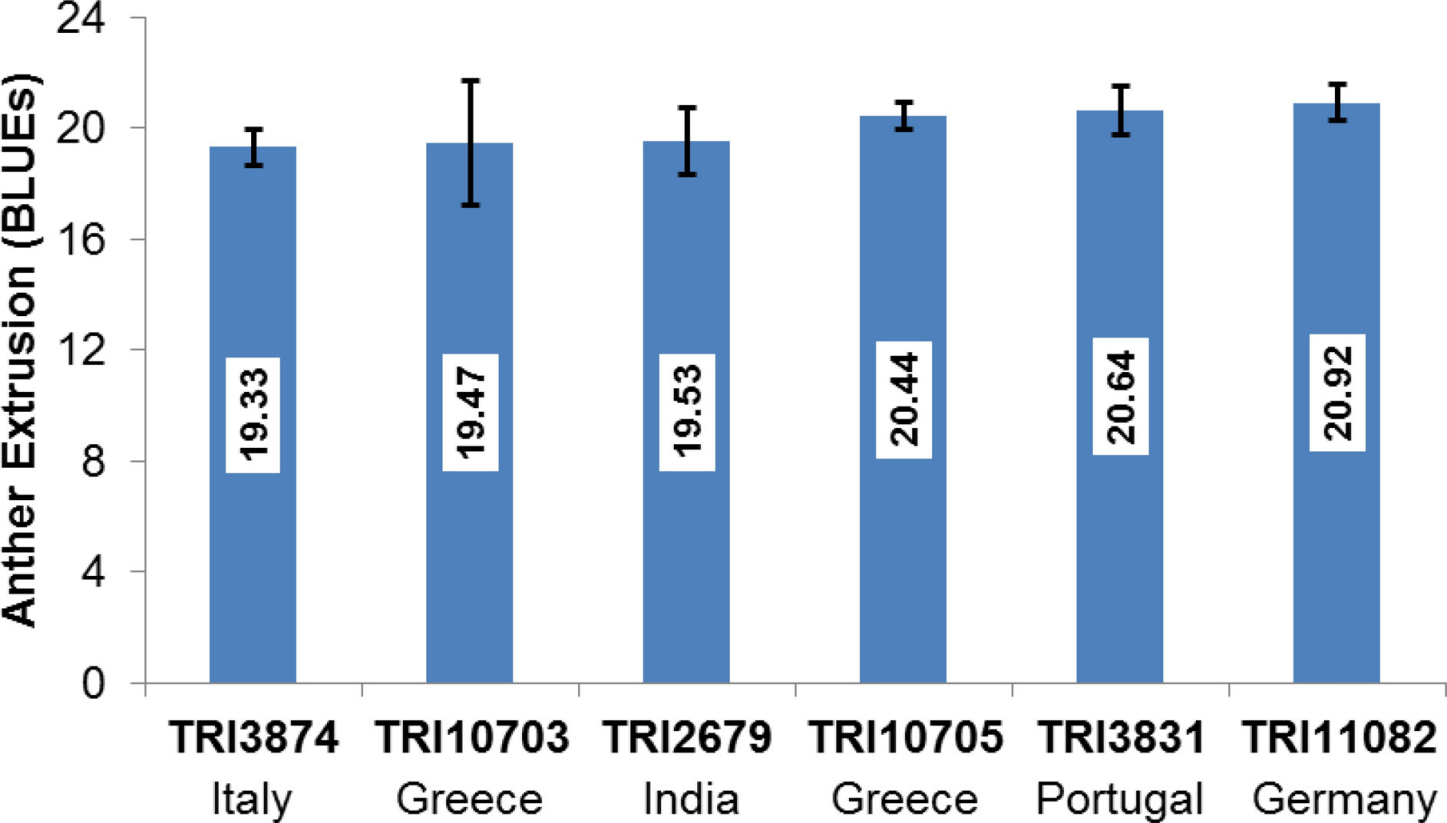
AE performance of the top six accessions (AE >80%) based on BLUE values ±SE. The name of the country indicated below the accessions stands for the country of origin of the accessions. Numbers on the Y-axis represent the number of extruded anthers.

Anther extrusion (AE) was scored for 111 accessions and showed a wide range of variation among the genotypes and the years. Each of the 111 accessions according to the country of origin and their AE scores are given in S1 and S2 Tables. For the year 2015, two methods to measure the AE were adopted *i*.*e*., direct scoring of the anthers in the field as in the years 2013 and 2014 and collecting and freezing the spikes and determining the AE in the lab. The two methods used for scoring in 2015 were strongly correlated with one another (r = +0.81, P <0.001). The laboratory-based method used in 2015 was preferred for the subsequent genetic analysis, largely because it avoided problems associated with the lodging of the many non-semi-dwarf accessions. AE scores varied markedly between the accessions. The years 2013, 2014 and BLUE values were close to a normal distribution, however some skewing was observed in the year 2015 which may be due to environmental conditions (S1 Fig). The minimum BLUE value across all three growing years was 2.94, the maximum was 20.92 and the mean was 13.24 (S1 Fig and S2 Table). The observed range in AE was comparable in the 2013 and 2014 trials (mean values of 10.18 and 10.58, respectively), while the 2015 experiment produced the much higher mean of 19.02 (Fig 1) which could be attributed to environment. The extent of the AE correlation among growing years and BLUE values ranged from r = +0.56 (2013 *vs* 2015) to 0.91 (2014 *vs* BLUE values) (Fig 2). The level of repeatability among biological replicates was 0.95 for 2013 and 2014, and 0.97 for 2015. The broad sense heritability was 0.84 across all three years. Six accessions, displayed an AE >80% with AE BLUE values ranging from 19.33 to 20.92 (S2 Table), and the trait was highly stable across years; five of these cultivars were bred in Europe and one in India (Fig 3).

### The distribution of DArT markers, linkage disequilibrium and the presence of population structure

The imposition of the minor allele frequency (MAF) threshold >5% reduced the DArT marker set by 261. Of the remaining 2,575 markers, 2,166 had a known map location, which spread unevenly across all three constituent genomes; however, each of the 21 chromosomes was represented. The B-genome had the highest coverage (43.6% of the markers), while 37.7% mapped to the A genome and 18.7% to the D genome; 15.2% of the markers mapped to one of the three homoeologous group 1 chromosomes, while only 6.3% mapped to a homoeologous group 4 chromosome. The single most populated chromosome was 3B (205 loci) and the least populated was chromosome 4D (eight loci); the mean number of loci per chromosome was 103.1 (S2 Fig).

Intra- and inter-chromosomal LD assessment was performed using pair-wise DArT loci. A considerable variability was seen in chromosome-wise analysis of LD (S3 Fig). On average, the significant levels of LD (*r*^*2*^ declining below 0.2) extended for ca. 2.5 cM. As the least number of markers was mapped on the D-genome, for some D-genome chromosomes no LD could be calculated. Intra-chromosomal locus pairs had a higher mean *r*^*2*^ value (0.05) than inter-chromosomal locus pairs (0.01) (S3 Table).

Our Structure results using the DArT markers with MAF >5% indicated the ΔK peak at K = 2, providing support for the existence of two genetically distinct sub-groups in our association panel. Structure results are grouped and graphed with CLUMPAK (S4A Fig). Both Structure Harvester and CLUMPAK exhibited the same results. ΔK and the mean likelihood values are plotted in S4B Fig.

**Fig 4:**
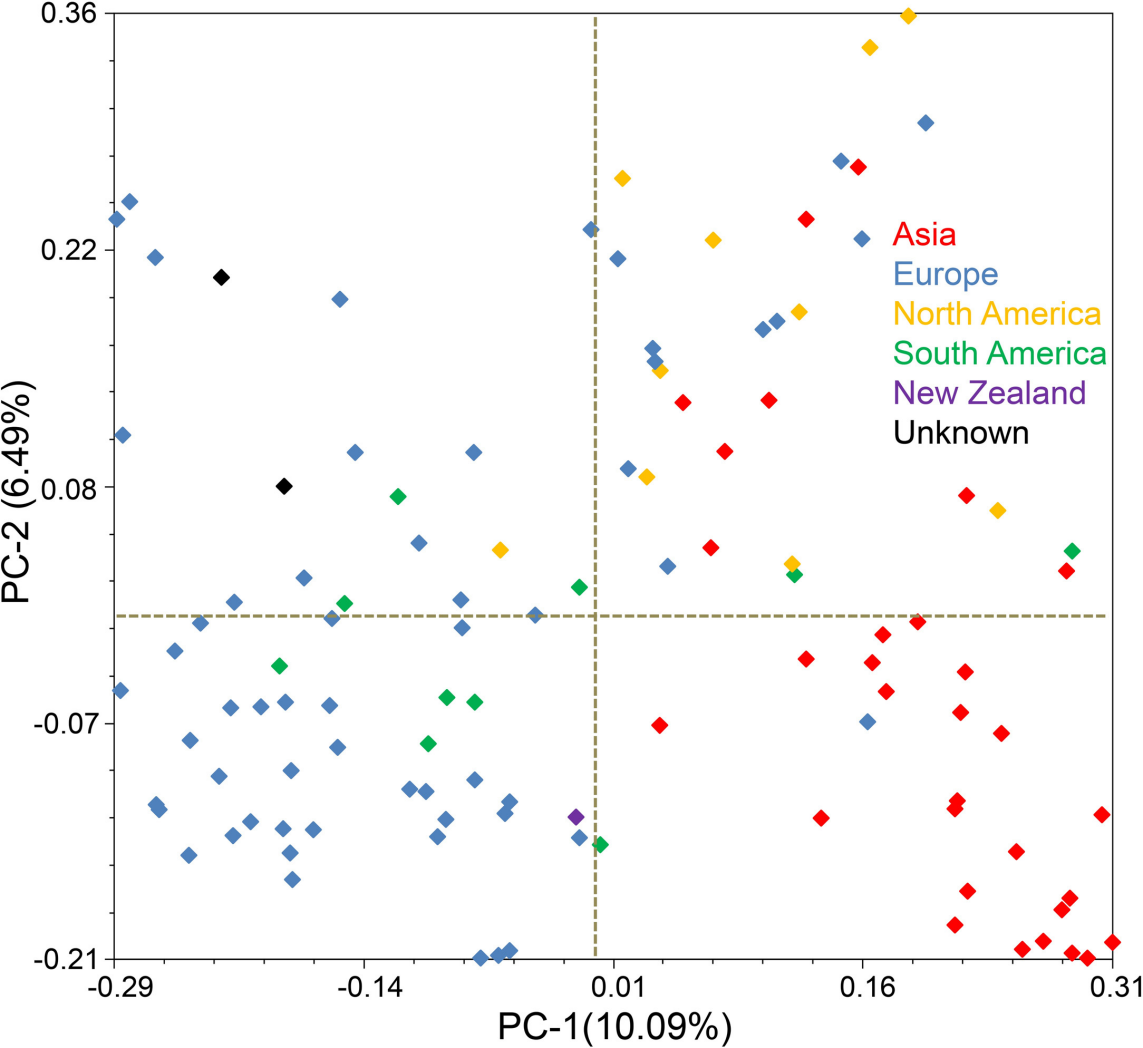
Principal coordinate analysis of the AM germplasm panel based on DArT genotype. The cultivars bred in both Europe and Asia form recognizable clusters, but those bred in the Americas do not. Colour code is given in the figure.

A PCoA-based test for the existence of clustering among accessions is illustrated in Fig 4. The PCoA analysis was generally consistent with the Structure results. In order to simplify the PCoA plot, we highlighted the marks with different colours to represent different continents from where the accessions had been taken. A clear distinction could be drawn between the European and Asian accessions, while there was little evidence of any clustering for the American ones.

### Allelic variation at major genes

The allelic status of the genes underlying photoperiod sensitivity (*Ppd-D1*) [31], vernalization requirement (*Vrn-1* (*Vrn-A1*, *-B1* and *-D1*)) [32] and semi-dwarfism (*Rht-1* (*Rht-B1* and *-D1*)) [33] was obtained by PCR assays. The observed allele frequencies at these genes are given in S2 Table. The insensitive allele at *Ppd-D1* was carried by 16 accessions. The recessive allele at the three *Vrn-1* homoeoloci were present in, respectively, 75, 37 and 98 of the cultivars; although the expectation is that a spring type should carry the dominant allele at least at one of the three loci, six cultivars carried the recessive allele at all three loci (S2 Table), a genetic constitution which generally imposes a vernalization requirement [32]. Since most of the accessions had been released in 1970’s, the frequency of the semi-dwarfing allele at both *Rht-1* loci was low (three accessions were the mutant genotype *Rht-B1b* and one was *Rht-D1b*). The accession TRI 10296 was a double dwarf (*Rht-B1b* / *Rht-D1b*), and its mean plant height across the three growing seasons was only 67.0 cm. We did the MTA analysis based on candidate gene genotypes and found no significant MTA for these major genes.

### Marker trait associations

**Fig 5:**
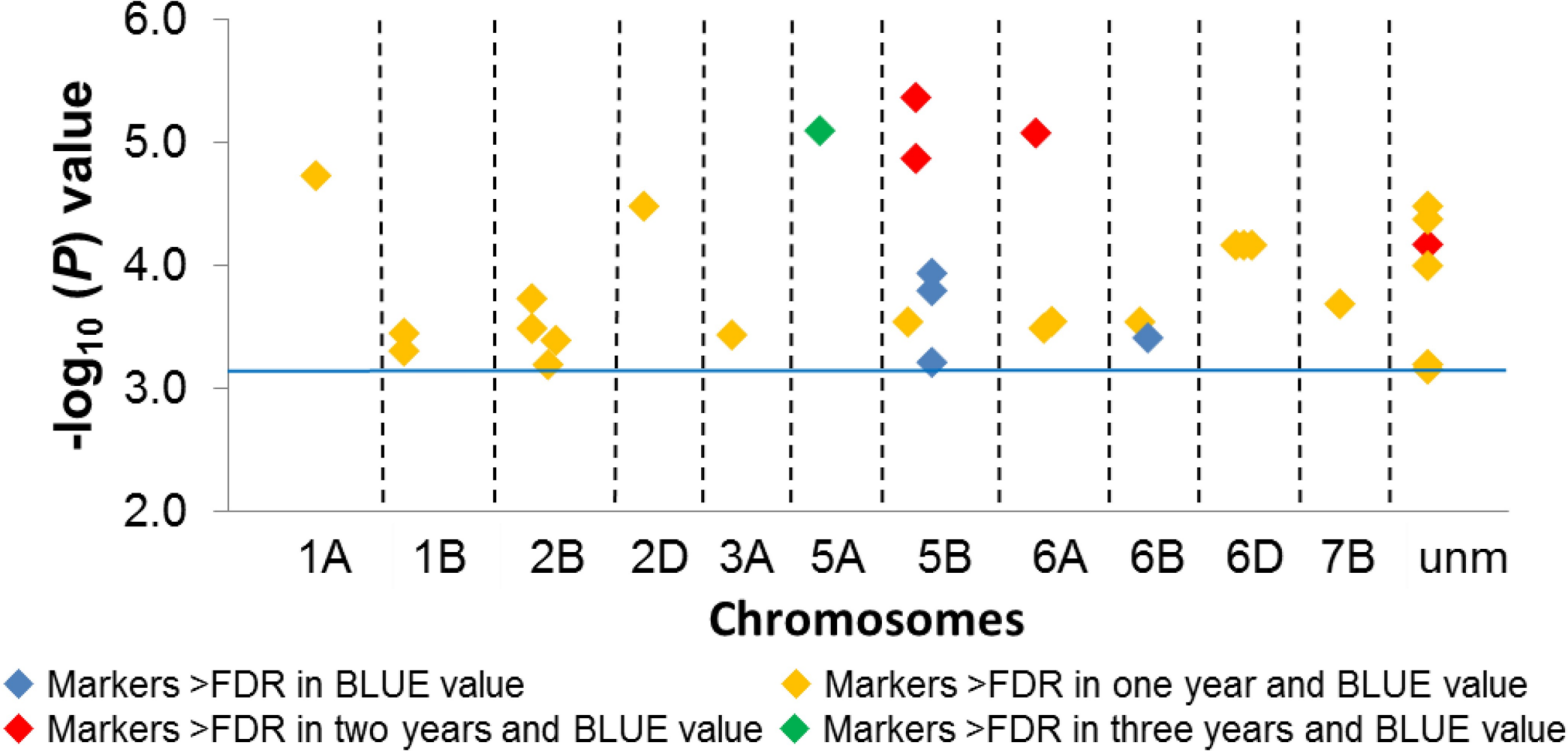
Manhattan plot illustrating the chromosomal distribution of the 31 significant AE markers (|log_10_ (*P*)| >FDR) based on BLUE values. The blue line marks the FDR threshold. unm stands for unmapped markers. Colour code for consistently significant markers is given in the figure.

**Fig 6:**
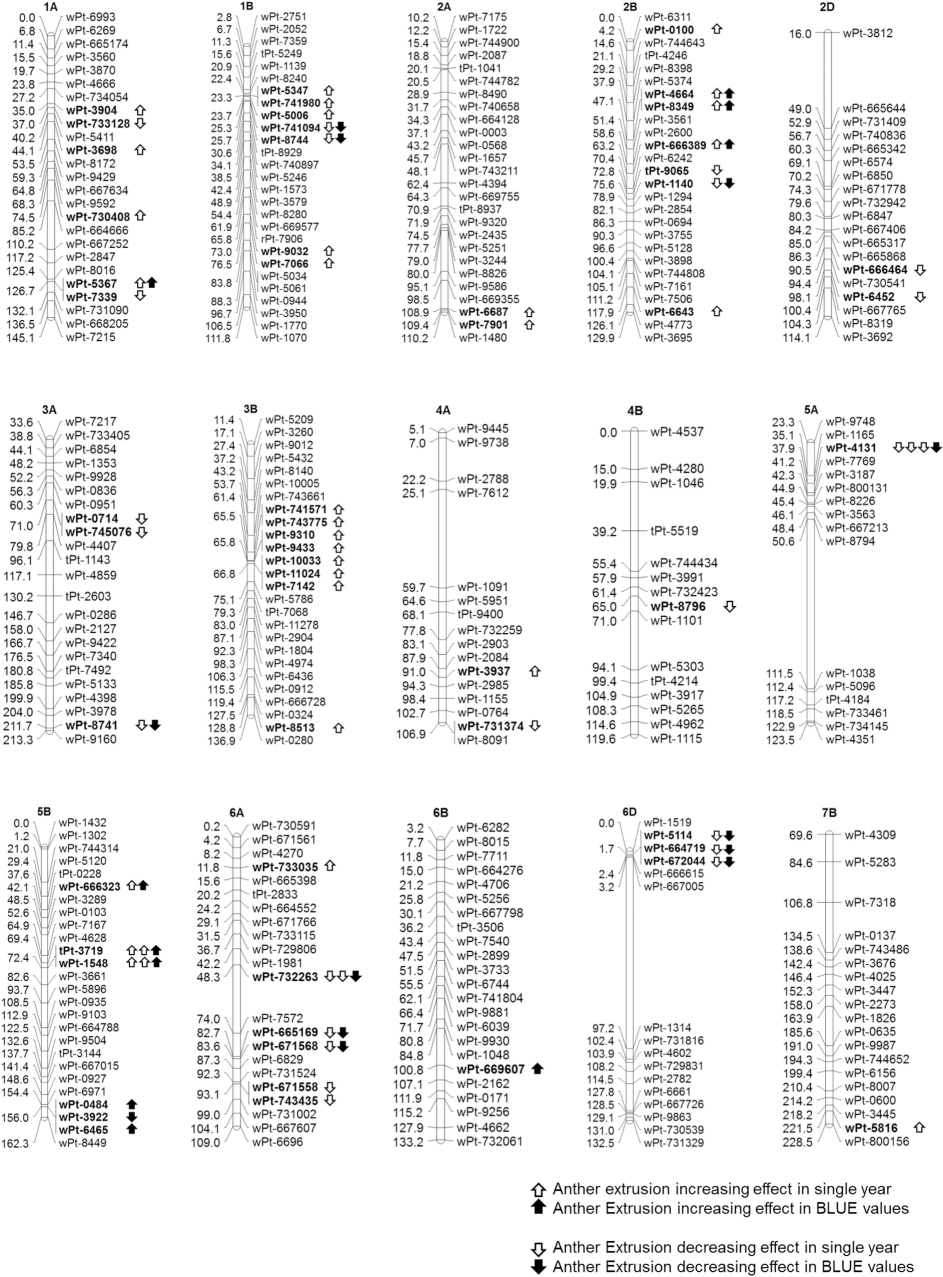
Linkage maps of the chromosomes harbouring significant AE MTAs.

The Q-Q plots, which compare observed *vs* expected |log_10_ (*P*)| values, indicated the most stringent analysis for the year 2014 among all years and their BLUE values (S5 Fig). In total, 131 significant MTAs (|log_10_ (*P*)| >FDR), involving 99 DArT markers were identified across the three years and BLUE values (S4 Table). Of these 131 significant MTAs, 31 were based on BLUE values (involving 31 markers) (Table 1 and Fig 5). The chromosomal distribution and location of all mapped MTAs are shown in Fig 6. Of the significant MTAs, 73 were associated with just one of the three years or BLUE value, 42 (based on 21 markers) were present in one year and BLUE value, 12 (based on 4 markers) in two years and BLUE value and four (one marker) in all three years and BLUE value (S5 Table). The B-genome harboured 48 MTAs, the A genome 29 and the D genome 23.

**Table 1:** Significant markers based on BLUE values.

Marker	2013	2014	2015	BLUE value	Chromosome	Position (cM)	-log_10_ (*P*) BLUEs	Effect BLUEs	% R^2^ BLUES
wPt-5367			✓	✓	1A	126.674	4.733	1.76	4.58
wPt-8744			✓	✓	1B	25.664	3.308	-1.75	5.42
wPt-741094			✓	✓	1B	25.268	3.452	-1.83	5.05
wPt-4664	✓			✓	2B	47.076	3.492	1.32	4.92
wPt-8349	✓			✓	2B	47.076	3.734	1.39	5.33
wPt-666389			✓	✓	2B	63.226	3.198	1.32	3.64
wPt-1140			✓	✓	2B	75.561	3.394	-1.94	3.25
wPt-9997			✓	✓	2D	unm	4.486	2.87	6.49
wPt-8741	✓			✓	3A	211.696	3.439	-2.00	4.48
wPt-4131**	✓	✓	✓	✓	5A	37.918	5.100	-1.69	8.61
wPt-666323			✓	✓	5B	42.133	3.545	1.75	7.26
tPt-3719**		✓	✓	✓	5B	72.410	4.873	1.72	4.29
wPt-1548**		✓	✓	✓	5B	72.410	5.369	1.88	4.53
wPt-0484				✓	5B	155.960	3.940	1.58	3.64
wPt-3922				✓	5B	155.960	3.216	-1.44	3.06
wPt-6465				✓	5B	155.960	3.798	1.54	3.51
wPt-732263**		✓	✓	✓	6A	48.289	5.080	-2.67	4.88
wPt-665169		✓		✓	6A	82.745	3.493	-1.33	7.46
wPt-671568		✓		✓	6A	83.649	3.548	-1.36	8.64
wPt-669607				✓	6B	100.799	3.415	1.51	5.66
wPt-6160	✓			✓	6B	unm	3.545	2.70	4.91
wPt-5114			✓	✓	6D	1.676	4.169	-1.59	4.01
wPt-664719			✓	✓	6D	1.676	4.169	-1.59	4.01
wPt-672044			✓	✓	6D	1.676	4.169	-1.59	4.01
wPt-2737			✓	✓	7B	unm	3.690	2.46	2.28
wPt-1695			✓	✓	unm	unm	4.174	-1.61	4.16
wPt-665961			✓	✓	unm	unm	3.198	3.33	1.66
wPt-666223			✓	✓	unm	unm	4.486	2.87	6.49
wPt-666857			✓	✓	unm	unm	3.160	2.38	2.40
wPt-741749**		✓	✓	✓	unm	unm	4.378	2.48	5.22
wPt-798333				✓	unm	unm	4.000	-1.49	5.50

✓ Markers above FDR value.

** Consistently significant markers appearing across at least two years and BLUE values (|log_10_ (*P*)| >FDR).

unm Unmapped markers.

To increase confidence in our new method to measure the AE in lab, we did MTA analysis on AE data taken from field and in lab for the year 2015, separately. In total, 49 marker-AE associations were found for the field-based method (S6A Table), while 71 for the lab-based method (S6B Table) at a threshold of (|log_10_ (*P*)| >3.0). In total, 42 of the 49 MTAs (85.71%) noted in the field-based method for AE measurement also appeared in the lab-based method (S6C Table).

Of all the 99 significant DArT markers (|log_10_ (*P*)| >FDR), a favourable effect (increasing effect on AE) was associated with 72 of the significant markers (S7A Table) and an unfavourable (decreasing effect on AE) with 27 (S7B Table). The distribution of these significant markers was different across accessions. Each cultivar carried a mixture of favourable alleles (1–70, mean = 45.30) and unfavourable (1–27, mean = 8.74) alleles. A maximum number of nine accessions harboured 60 or 64 favourable alleles each (S6A(i) Fig), while a maximum of twelve accessions harboured eight unfavourable alleles (S6A(ii) Fig). The accessions carrying the highest number of favourable alleles (70) were TRI10705 and TRI3633 (these accessions harboured one and three unfavourable alleles, respectively). One accession TRI10705 ranked among the six best accessions on the basis of phenotypic BLUE values (Fig 3), with TRI3633 also showing a relatively high amount of AE (71.54%). The linear regressions of the number of favourable alleles per plant versus the AE-BLUE values resulted in Y = 9.4 + 0.085X with an R^2^ = 0.172 (P <0.001) (S6B(i) Fig), while for the number of unfavourable alleles it was Y = 17.2–0.449X with R^2^ = 0.395 (P <0.001) (S6B(ii) Fig). This means varieties with more favourable and less unfavourable alleles have higher AE and the effects are partially additive.

**Fig 7:**
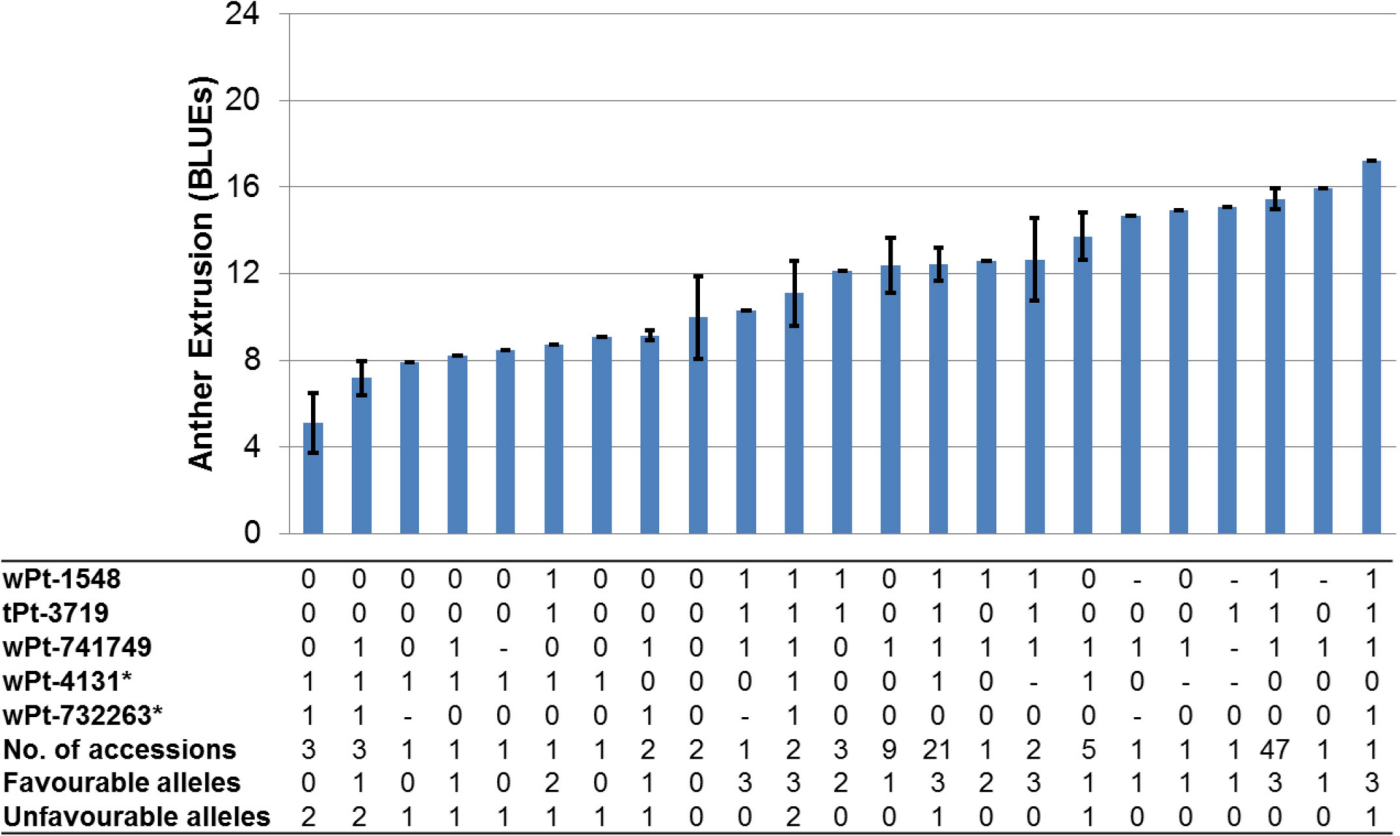
Clustering of accessions based on the pattern and number of favourable and unfavourable alleles at the consistently significant loci (|log_10_ (*P*)| >FDR). BLUE values of clusters of cultivars harbouring three favourable and zero unfavourable alleles exceed the mean phenotypic BLUE value. Asterisks mark the markers which reduce AE while “1” codes for favourable, “0” for unfavourable and “-” for missing alleles.

Of the 31 significant markers based on BLUE values (Table 1), 17 had a favourable effect on AE and 14, an unfavourable. Five markers were significantly associated across at least two years and BLUE values and hence were termed as consistently significant markers. These five markers were present on chromosomes 5A (37.92 cM), 5B (72.41 cM) and 6A (48.29 cM). One marker was unmapped, while two markers on chromosome 5B were co-segregating. The average phenotypic variance imparted by these consistently significant markers ranged from 4.29–8.61% (Table 1). Of the five consistently significant markers, three harboured favourable effects, while two had unfavourable effects. A clustering of the accessions, based on the allelic content at the five consistently significant markers, is given in Fig 7. The number of accessions carrying the most favourable and least unfavourable alleles had a greater than average AE score.

## Discussion

### Variation and heritability of anther extrusion

Anther Extrusion (AE) behaved as a quantitative trait among the germplasm panel, with each consistently significant marker (appearing significant across at least two years and BLUE values) contributing a modest proportion of phenotypic variance (R^2^ = 4.29–8.61%). A similar conclusion has been reached following a conventional QTL analysis [10], although it has also been suggested that the trait is governed by just a small number of genes [34]. The proportion of the phenotypic variance explained by the full set of consistently significant markers based on their BLUE values was 27.53%. Somewhat greater values have been reported by using the conventional QTL studies [9,10,35]. This small level of phenotypic variation can be attributed to the very complex nature of this trait, the environment and the low marker density especially on D-genome due to which some QTL might have been missed. The levels of repeatability among biological replicates were 0.95 for 2013 and 2014 while 0.97 for 2015. The broad sense heritability of AE was very high (0.84) among the three years (2013, 2014 and 2015). This high level of heritability is comparable to those of previous studies [9,10,35,36]. Nevertheless, high cross-environment levels of heritability for AE in wheat have been reported repeatedly in the literature [9,35,36].

### The relationship of AE with other traits

A variety of floral characteristics, including the timing of floret gaping, the length of the anthers and filaments, the size of the glumes, the separation between adjacent florets and the angle subtended between opposite florets are all documented as influencing the expression of AE [1,2,4]. Pollen mass is also highly correlated with AE [8]. Plant height is of importance in the production of F1 hybrid seed, since it is desirable to use a taller plant as the pollen donor in order to maximize the chances of cross fertilization [4]. Similarly, the flowering time of the pollen donor and male sterile parents must be compatible. A trait which has been directly associated with AE is resistance to the disease Fusarium head blight (FHB); cultivars which have high anther retention tend to be quite susceptible to FHB [9,35,37].

### Use of DArT markers, genome coverage and linkage disequilibrium

The use of DArT markers ensured a reasonable level of genome coverage, which is an important requirement for any association mapping experiment. The same class of marker has been exploited in a number of current and recent wheat mapping studies [9,10,16–19,27,35,38], reflecting the good level of reliability and cost-effectiveness of this marker platform. Coverage was much higher for the B-genome chromosomes than for the D-genome ones, mirroring the long established pattern which has emerged from the use of other DNA-based marker types, such as RFLPs and microsatellites, and confirming the outcomes of similar investigations reported by Bordes et al. [27] and Crossa et al. [38]. One consequence of the relatively low level of marker informativeness displayed by the D-genome chromosomes is a comparative foreshortening of the length of their linkage maps [39]. The likely reason for this pronounced low level of marker polymorphism is that the allopolyploidization event which introduced the D-genome into tetraploid emmer wheat occurred relatively recently [40], while the B genome by A genome hybrid is thought to date back around 0.5 million years [41]; thus much more time has been available for the build-up of variation through mutation in the A and B genomes.

The power of association mapping which determines the effectiveness of marker assisted selection (MAS) may be significantly affected by the extent of LD across chromosomes [42]. Reduced diversity in hexaploid wheat as a consequence of polyploidization and selection pressure are proposed as indications that changes the LD across chromosomes [43,44]. Normally LD is not constant across the genome [45] and varied levels of LD decay/chromosome have been reported in previous studies [38,45,46]. In our studies, chromosome-wise LD varied markedly, particularly in the D-genome which may be due to the low marker coverage of D-genome.

### The location of consistently significant markers

Significant AE-MTAs were identified on six of the seven A genome chromosomes (not 7A), all B-genome chromosomes and on three of the D-genome chromosomes (1D, 2D and 6D). The continuous distribution of AE in our study and previous results suggest that several factors are involved in the inheritance of this trait [9,10,35,36]. The consistently significant markers are considered important in our analysis. All of these markers and their locations are different from the previous studies which largely relied on the bi-parental populations. Chromosome 5A featured an unfavourable consistently significant MTA associated with marker *wPt-4131* present at 37.9 cM position and explained 8.61% of the mean phenotypic variation. The same chromosome has been implicated as the site for an AE-QTL, although not in the same region [35], while Lu et al. [9] have identified loci controlling both deoxynivalenol (a fungal toxin) content and kernel damage caused by FHB in the same region. Two consistently significant favourable co-segregating sites were detected on chromosome 5B (72.41 cM), one associated with *tPt-3719* and the other with *wPt-1548*. Although Lu et al. [9] have described a putative AE-QTL on this chromosome; its location does not match the present associated markers. The consistently significant MTA on chromosome 6A (48.29 cM) associated with *wPt-732263* acted to reduce AE. An important QTL for AE in a doubled haploid population was detected by Skinnes et al. [10] on the short arm of the same chromosome. This QTL explained 15.6% of the mean phenotypic variance and mapped in the confidence interval of 3.2–7.2 cM of chromosome 6A. Based on the mapping position it is a different QTL than the QTL detected by *wPt-732263*. The consistently significant marker *wPt-741749* was unmapped in our data. In the literature, it was located onto the consensus map of durum wheat (Meridiano X Claudio) on chromosome 1B at 12.2 cM position by Maccaferri et al [47]. Skinnes et al. [10] have reported a major AE-QTL on this chromosome in the confidence interval of 86–102 cM explaining a mean phenotypic variance of 7.4%, which is based on the mapping location a different QTL from the one detected by *wPt-741749*. Recent literature reported on anther extrusion/retention relies mainly on recombinant inbred lines and doubled haploid lines for both spring [9,10] and winter wheat cultivars[35]. The germplasm used in these studies is very different based on origin and years of release from the accessions used in our analysis, which may be a reason that no coinciding QTL loci were discovered.

## Conclusions

The association mapping analysis has confirmed that anther extrusion (AE) in wheat is under the control of a number of gene loci, some of which act favourably and others unfavourably. As a result, there is potential for genetic advance for AE, by selecting for the former and against the latter. Thus, a breeding strategy based on marker assisted selection would need to incorporate methods of genomic selection [48,49], which is becoming possible by the continuing refinement in the means to achieve massively parallel genotyping. AE is an important trait in the context of F1 hybrid grain production. An understanding of its genetic basis will therefore serve to promote the exploitation of heterosis in this key crop, which still relies almost entirely on homozygous inbreds.

## Supporting Information

S1 Fig The phenotypic distribution of AE across growing years and BLUE values in members of the AM germplasm panel. (PDF)

S2 Fig The chromosome-by-chromosome, homoeologous group-by-homoeologous group and genome-by-genome distribution of the set of 2,575 DArT markers. unm stands for unmapped markers.
(PDF)

S3 Fig Intra and inter-chromosomal heat maps of linkage disequilibrium (LD) values. Heat maps depict *r*^*2*^ between markers. Intra-chromosomal plots of LD decay show *r*^*2*^ against the genetic distances (cM) between pairs of DArT loci in the spring wheat genome.
(PDF)

S4 Fig –(A). Structure results displaying continent-wise distribution of whole spring wheat panel based on DArT genotype as K = 2. The white lines mark the separation between the continents. (B) Structure harvester results displaying the plots of the (i) ΔK and (ii) log-likelihood values for the analysis based on DArT markers. K = 2 is the most appropriate.
(PDF)

S5 Fig Q-Q plots for each growing year and BLUE values, depicting expected *vs* observed |log_10_ (*P*)| values. (PDF)

S6 Fig –(A). The number of (i) positive and (ii) negative AE alleles harboured by individual cultivars. (B) Linear regression of BLUE values for AE against the number of (i) favourable and (ii) unfavourable AE alleles harboured by individual cultivars. Higher numbers of favourable alleles are associated with an increased BLUE value for AE, and higher numbers of unfavourable alleles with a decreased BLUE value for AE.
(PDF)

S1 Table The AM germplasm panel including their country of origin. (XLSX)

S2 Table Allelic variation across the AM germplasm panel, based on PCR amplicons obtained from *Ppd-D1*, three *Vrn-1* genes and two *Rht-1* genes. The AE performance in each growing year and the respective BLUE values are also given.
(XLSX)

S3 Table Intra and inter-chromosomal LD for loci pairs. (XLSX)

S4 Table The set of significant MTAs (|log_10_ (*P*)| >FDR). Columns G to L record the additive effects and the R2 for each year; columns M and N indicate the additive effects and the R2 of the BLUE values.
(XLSX)

S5 Table The set of significant AE-MTAs (|log_10_ (*P*)| >FDR). Those restricted to a single year or BLUE values numbered 73, those to one year and BLUE values numbered 42, those to two years and BLUE values numbered 12, and those to all three years and BLUE values numbered four.
(XLSX)

S6 Table (A). List of MTAs appeared in 2015 field based measurement of AE. (B). List of MTAs appeared in 2015 Lab based measurement of AE. (C). Comparison between two methods.
(XLSX)

S7 Table –(A). Number of favourable alleles per cultivar. A total of 72 marker alleles were associated with a favourable additive effect on AE. (B) Number of unfavourable alleles per cultivar. A total of 27 marker alleles were associated with an unfavourable additive effect on AE.
(XLSX)
