# Supplementary material for: Genome-Wide Association Mapping of Anther Extrusion in Hexaploid Spring Wheat
Source: PLoS One. 2016 May 18;11(5):e0155494. doi: 10.1371/journal.pone.0155494 (PMC4871436; doi:10.1371/journal.pone.0155494)
Supplement: S1 Fig — (PDF) [file pone.0155494.s001.pdf]

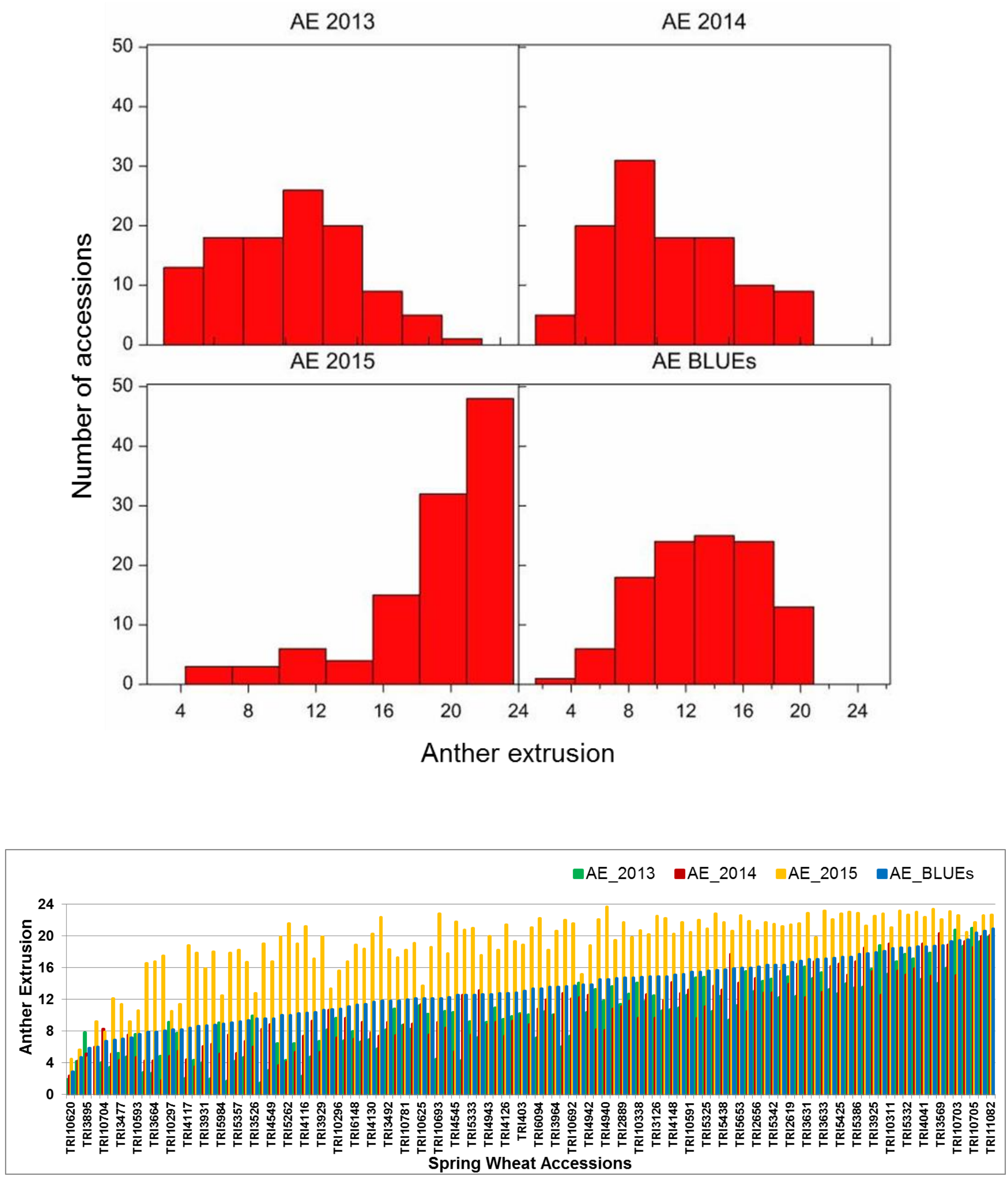

S1 Fig. The expression of AE across growing years and BLUE values in members of the AM germplasm panel.
