# Supplementary material for: Genome-Wide Association Mapping of Anther Extrusion in Hexaploid Spring Wheat
Source: PLoS One. 2016 May 18;11(5):e0155494. doi: 10.1371/journal.pone.0155494 (PMC4871436; doi:10.1371/journal.pone.0155494)
Supplement: S2 Fig — unm stands for unmapped markers. (PDF) [file pone.0155494.s002.pdf]

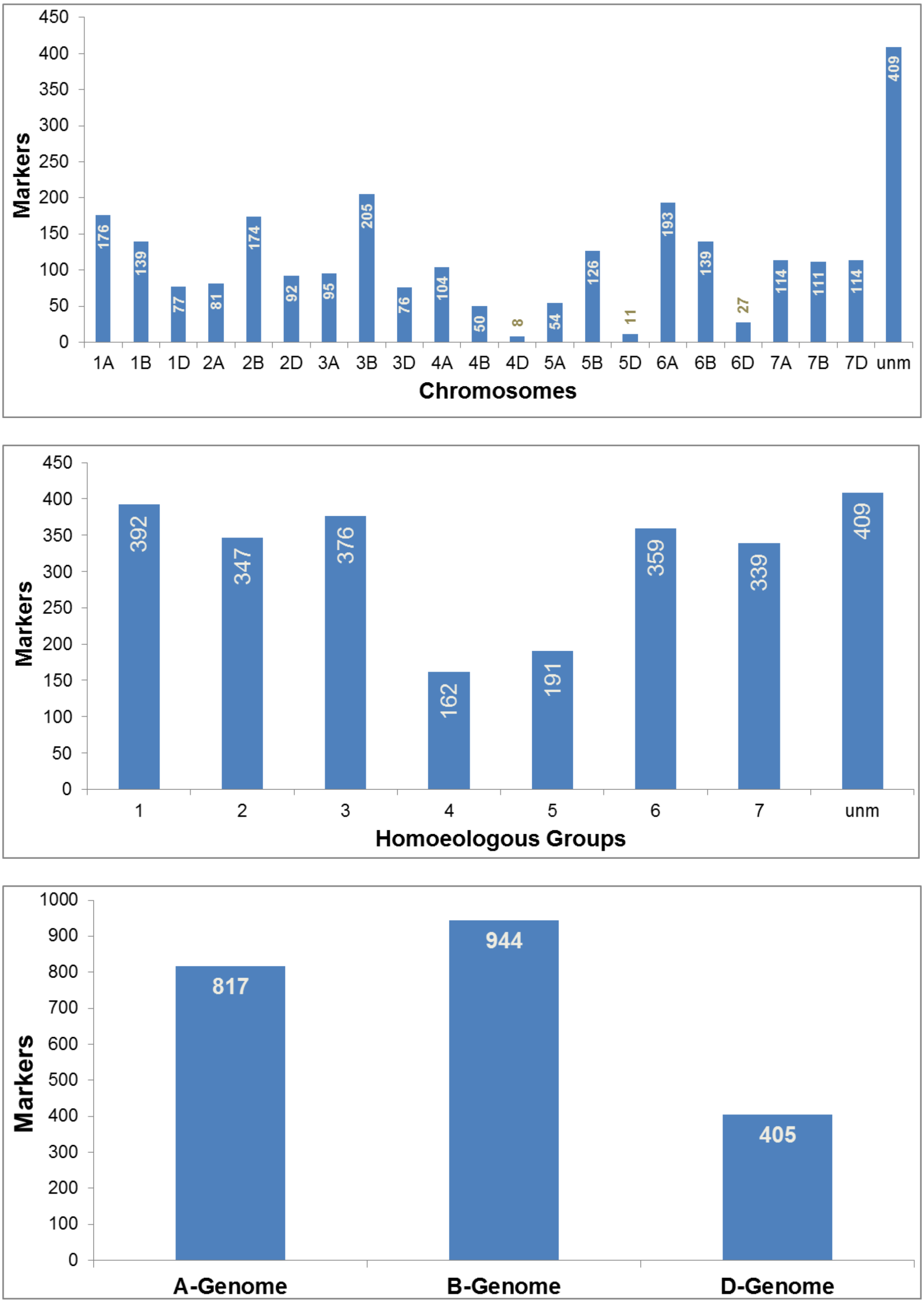

S2 Fig. The chromosome-by-chromosome, homoeologous group-by-homoeologous group and genome-by-genome distribution of the set of 2,575 DArT markers. unm stands for unmapped markers.
