# Supplementary material for: Genome-Wide Association Mapping of Anther Extrusion in Hexaploid Spring Wheat
Source: PLoS One. 2016 May 18;11(5):e0155494. doi: 10.1371/journal.pone.0155494 (PMC4871436; doi:10.1371/journal.pone.0155494)
Supplement: S3 Fig — Heat maps depict r2 between markers. Intra-chromosomal plots of LD decay show r2 against the genetic distances (cM) between pairs of DArT loci in the spring wheat genome. (PDF) [file pone.0155494.s003.pdf]

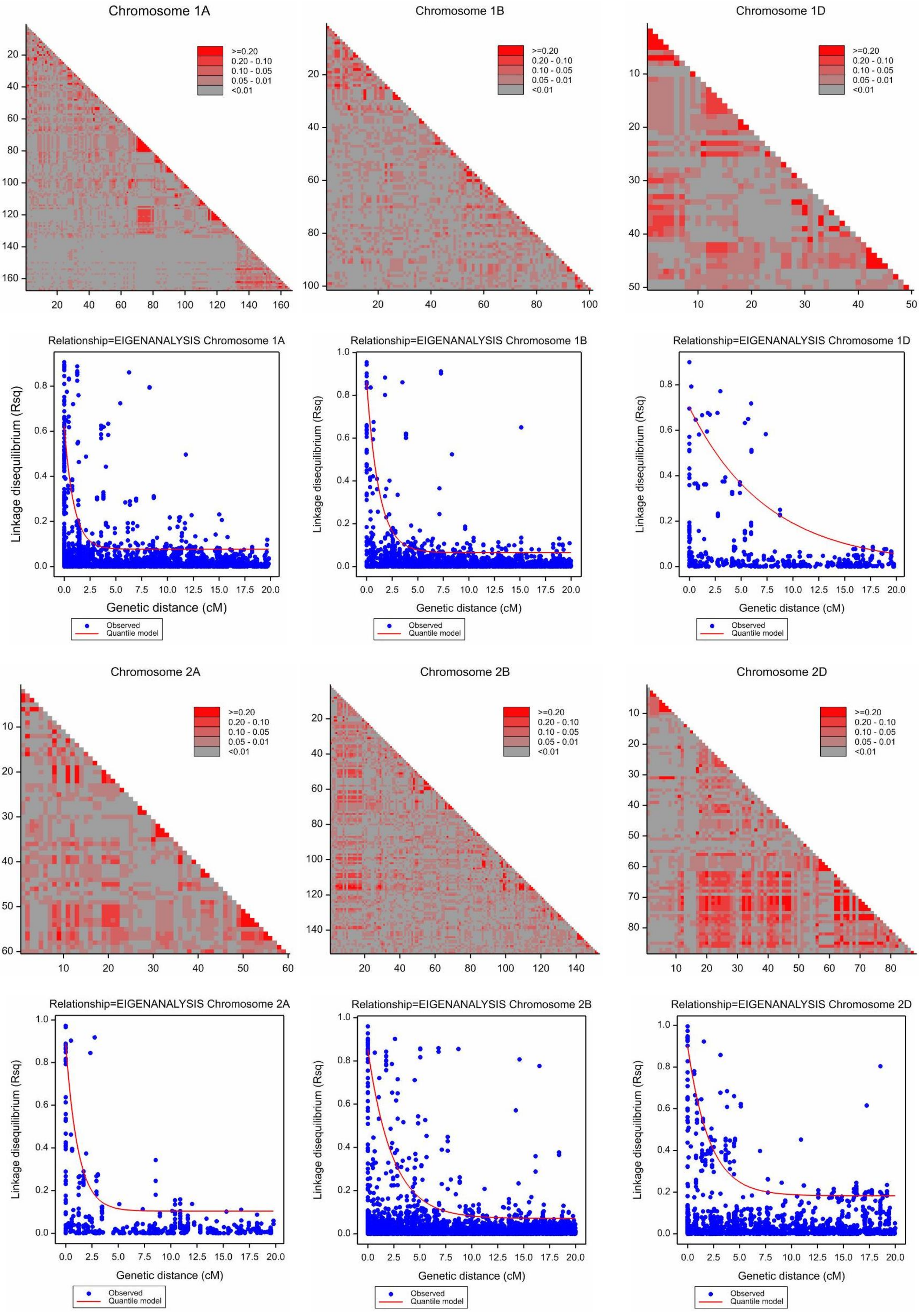
Chromosome 1A
Chromosome 1B
Chromosome 1D
>=0.20
0.20 - 0.10
0.10 - 0.05
0.05 - 0.01
<0.01
Relationship=EIGENANALYSIS Chromosome 1A
Relationship=EIGENANALYSIS Chromosome 1B
Relationship=EIGENANALYSIS Chromosome 1D
Linkage disequilibrium (Rsq)
Genetic distance (cM)
Observed
Quantile model
Chromosome 2A
Chromosome 2B
Chromosome 2D
Relationship=EIGENANALYSIS Chromosome 2A
Relationship=EIGENANALYSIS Chromosome 2B
Relationship=EIGENANALYSIS Chromosome 2D

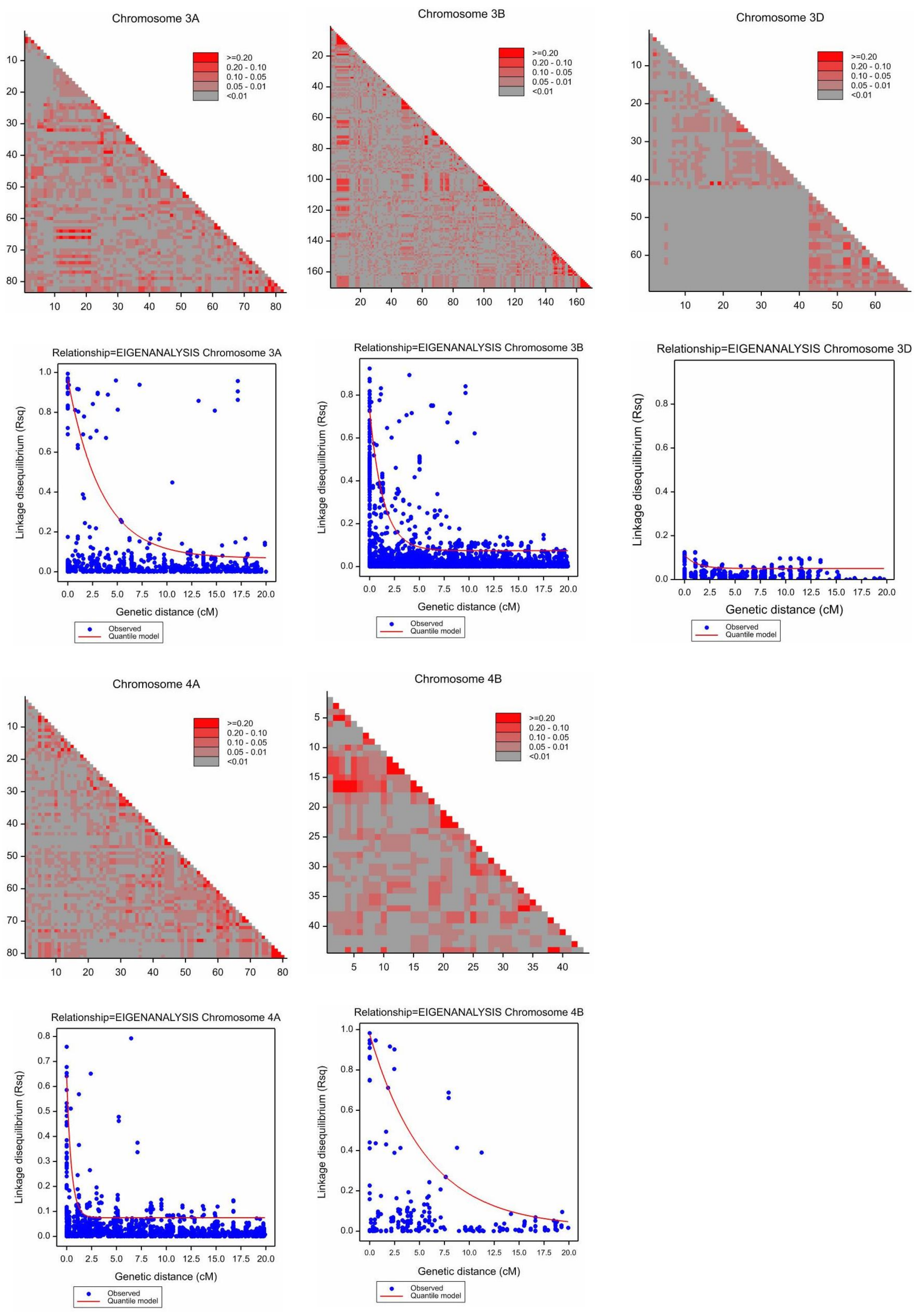

Chromosome 3A
Chromosome 3B
Chromosome 3D
>=0.20
0.20 - 0.10
0.10 - 0.05
0.05 - 0.01
<0.01
Relationship=EIGENANALYSIS Chromosome 3A
Relationship=EIGENANALYSIS Chromosome 3B
Relationship=EIGENANALYSIS Chromosome 3D
Linkage disequilibrium (Rsq)
Genetic distance (cM)
Observed
Quantile model
Chromosome 4A
Chromosome 4B
Relationship=EIGENANALYSIS Chromosome 4A
Relationship=EIGENANALYSIS Chromosome 4B

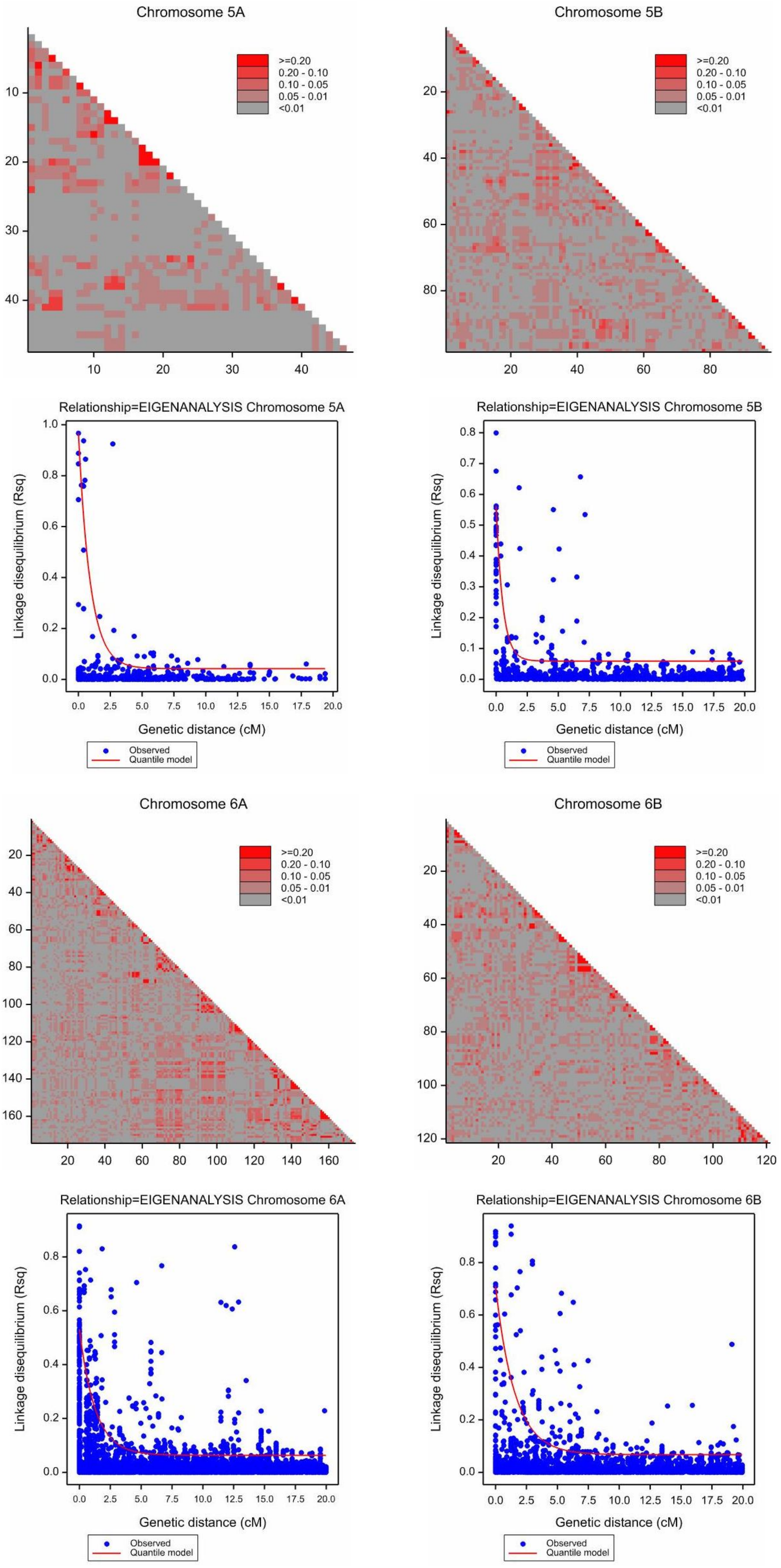
Chromosome 5A
>=0.20
0.20 - 0.10
0.10 - 0.05
0.05 - 0.01
<0.01
Chromosome 5B
Relationship=EIGENANALYSIS Chromosome 5A
Relationship=EIGENANALYSIS Chromosome 5B
Linkage disequilibrium (Rsq)
Genetic distance (cM)
Observed
Quantile model
Chromosome 6A
Chromosome 6B
Relationship=EIGENANALYSIS Chromosome 6A
Relationship=EIGENANALYSIS Chromosome 6B

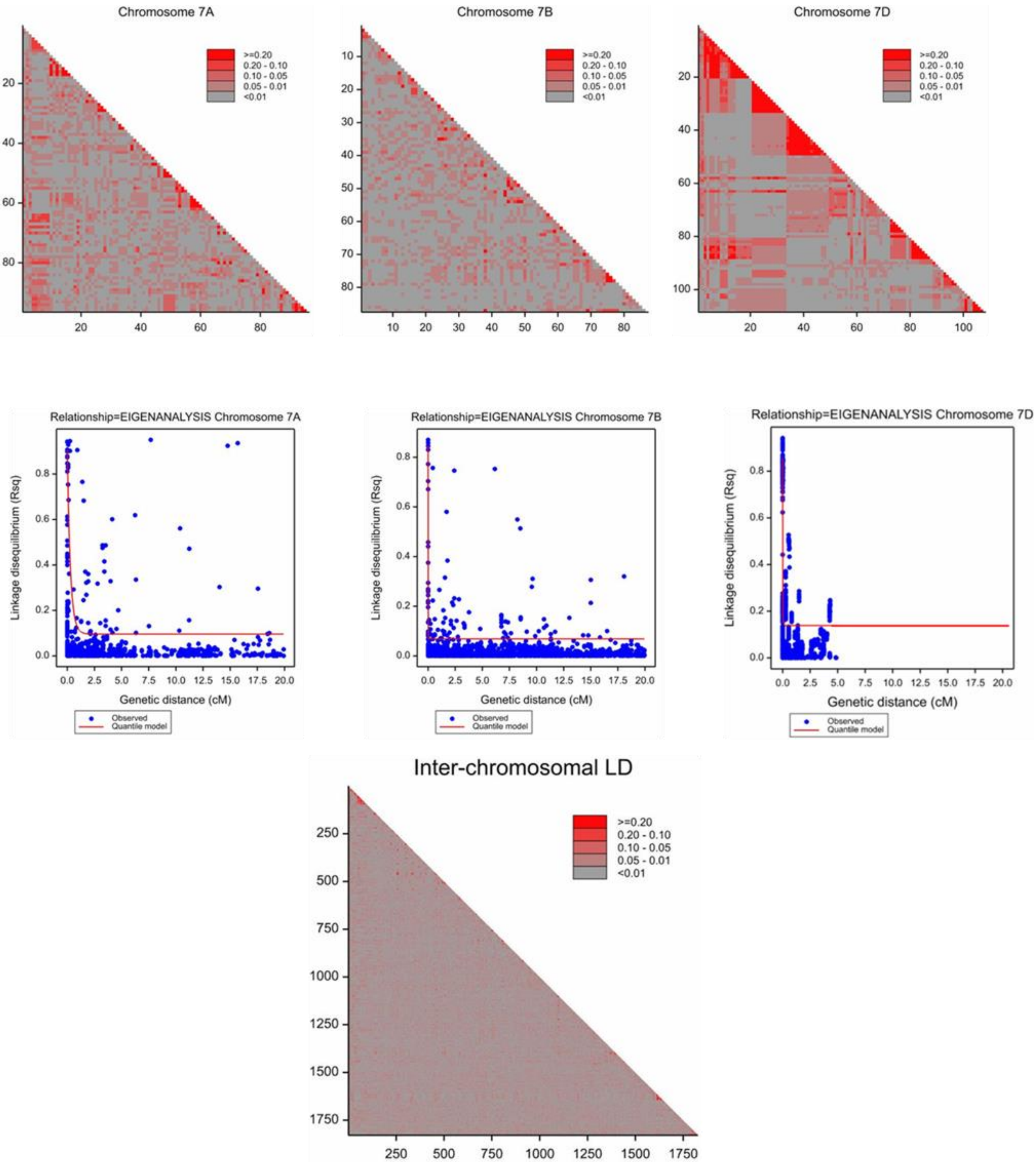

S3 Fig. Intra and inter-chromosomal heat maps of linkage disequilibrium (LD) values. Heat maps depict r^2 between markers. Intra-chromosomal plots of LD decay show r^2 against the genetic distances (cM) between pairs of DArT loci in the spring wheat genome.
