# Supplementary material for: Genome-Wide Association Mapping of Anther Extrusion in Hexaploid Spring Wheat
Source: PLoS One. 2016 May 18;11(5):e0155494. doi: 10.1371/journal.pone.0155494 (PMC4871436; doi:10.1371/journal.pone.0155494)
Supplement: S4 Fig — –(A). Structure results displaying continent-wise distribution of whole spring wheat panel based on DArT genotype as K = 2. The white lines mark the separation between the continents. (B) Structure harvester results displaying the plots of the (i) ΔK and (ii) log-likelihood values for the analysis based on DArT markers. K = 2 is the most appropriate. (PDF) [file pone.0155494.s004.pdf]

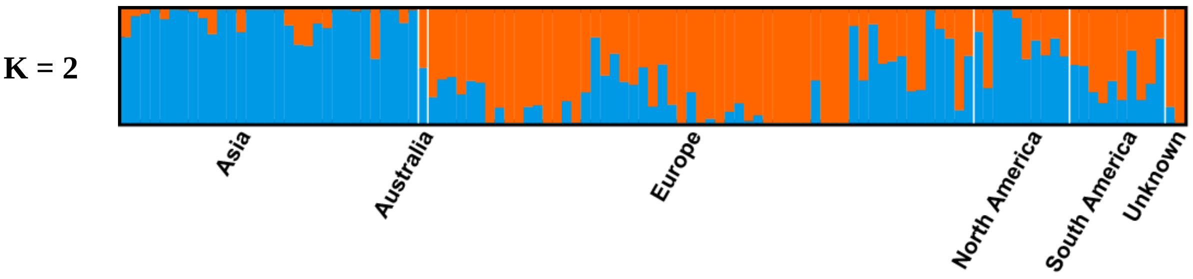

S4 Fig. – (A). Structure results displaying continent-wise distribution of whole spring wheat panel based on DArT genotype as K = 2. The white lines mark the separation between the continents.

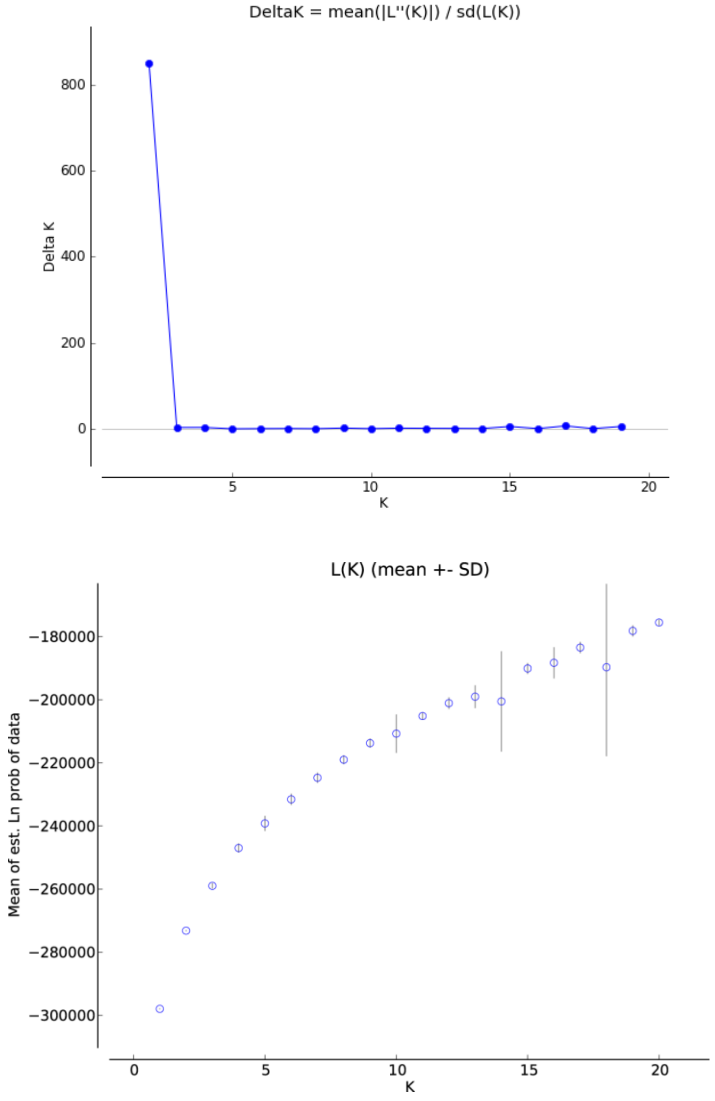

S4 Fig. – (B). Structure harvester results displaying the plots of the (i) ΔK and (ii) log-likelihood values for the analysis based on DArT markers. K = 2 is the most appropriate.
