# Supplementary material for: Genome-Wide Association Mapping of Anther Extrusion in Hexaploid Spring Wheat
Source: PLoS One. 2016 May 18;11(5):e0155494. doi: 10.1371/journal.pone.0155494 (PMC4871436; doi:10.1371/journal.pone.0155494)
Supplement: S5 Fig — (PDF) [file pone.0155494.s005.pdf]

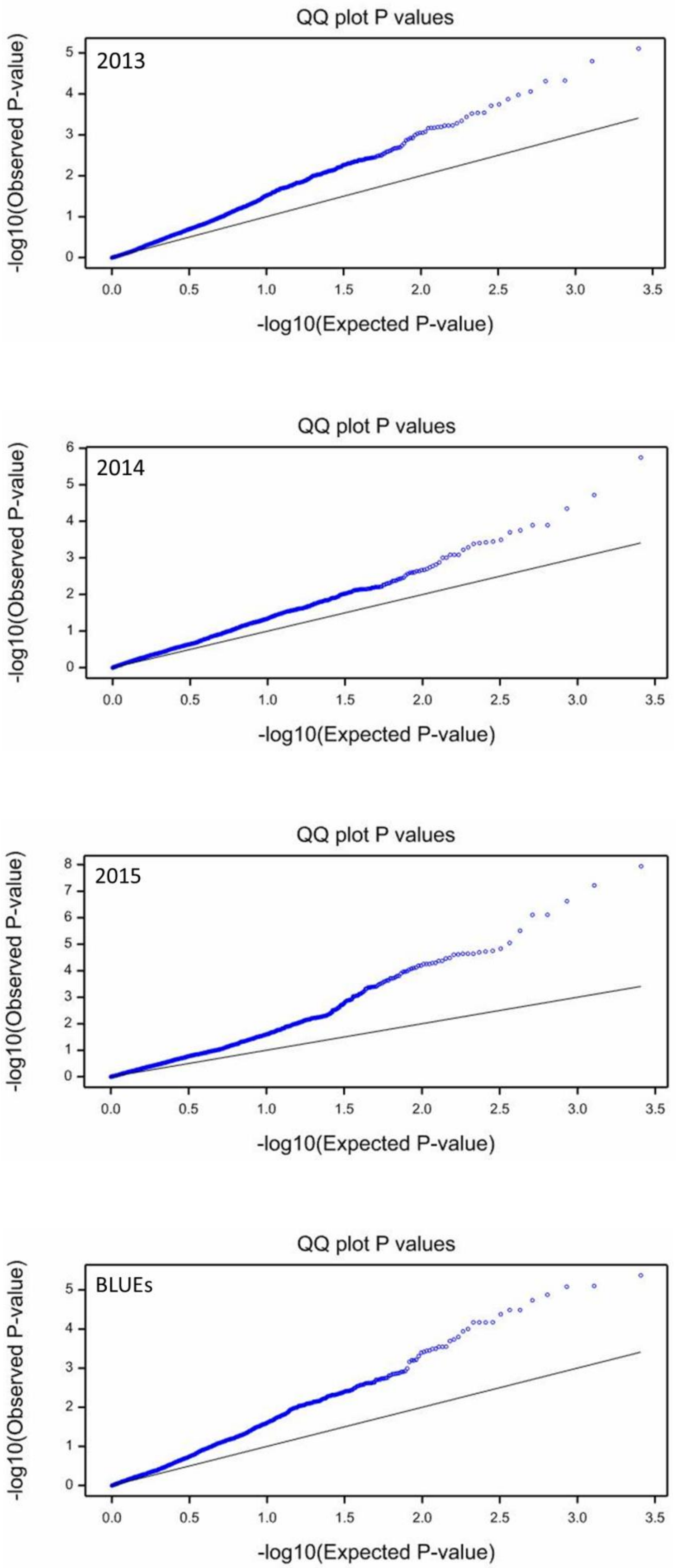

S5 Fig. Q-Q plots for each growing season and BLUE values, depicting expected *vs* observed $|\log_{10} (P)|$ values.
