# Supplementary material for: Genome-Wide Association Mapping of Anther Extrusion in Hexaploid Spring Wheat
Source: PLoS One. 2016 May 18;11(5):e0155494. doi: 10.1371/journal.pone.0155494 (PMC4871436; doi:10.1371/journal.pone.0155494)
Supplement: S6 Fig — –(A). The number of (i) positive and (ii) negative AE alleles harboured by individual cultivars. (B) Linear regression of BLUE values for AE against the number of (i) favourable and (ii) unfavourable AE alleles harboured by individual cultivars. Higher numbers of favourable alleles are associated with an increased BLUE value for AE, and higher numbers of unfavourable alleles with a decreased BLUE value for AE. (PDF) [file pone.0155494.s006.pdf]

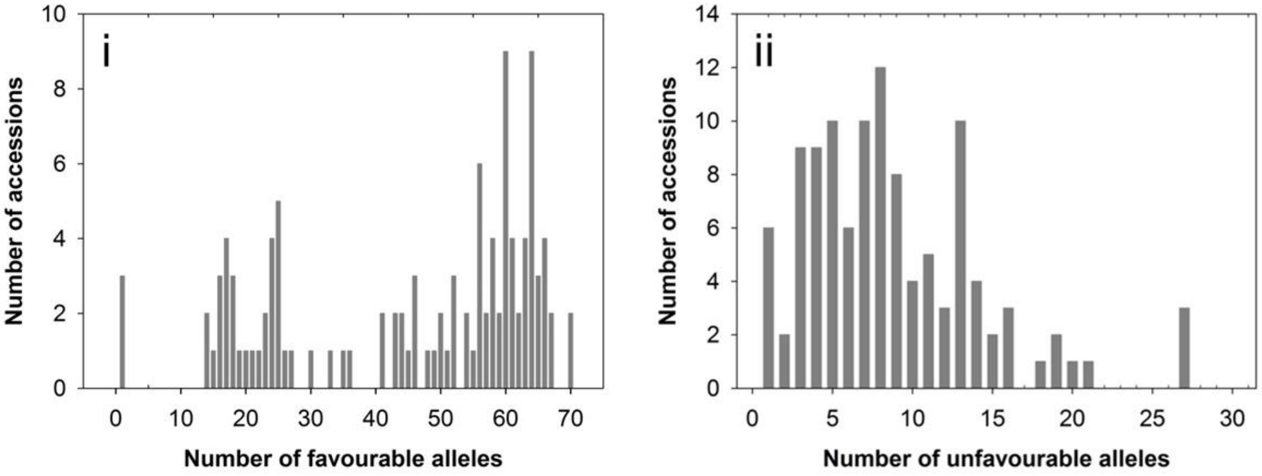

S6 Fig. – (A). The number of **(i)** positive and **(ii)** negative AE alleles harboured by individual cultivars.

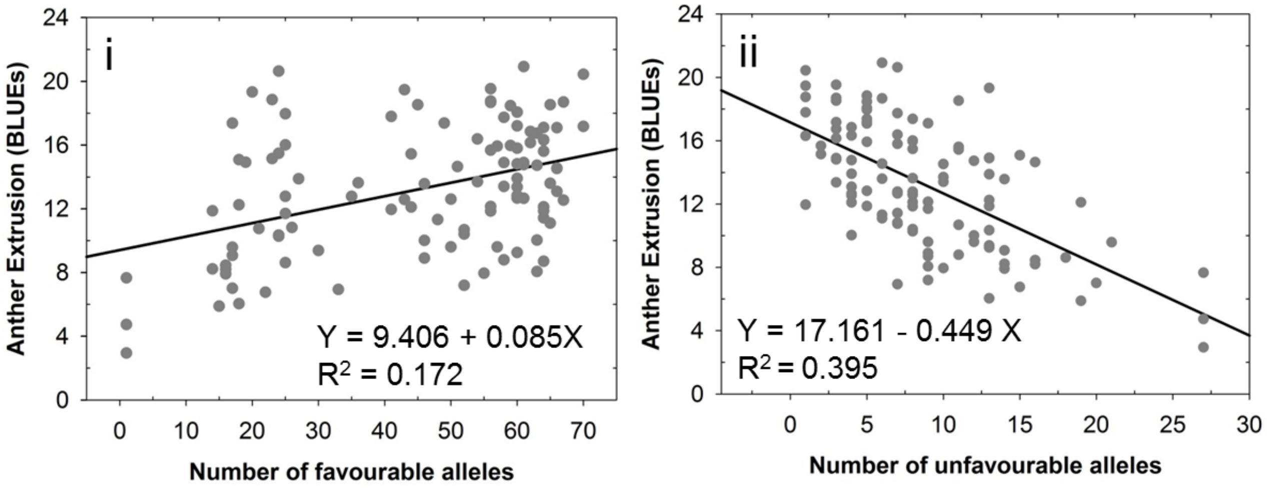

S6 Fig. – (B). Linear regression of BLUE values for AE against the number of **(i)** favourable and **(ii)** unfavourable AE alleles harboured by individual cultivars. Higher numbers of favourable alleles are associated with an increased BLUE value for AE, and higher numbers of unfavourable alleles with a decreased BLUE value for AE.
